# Western Mediterranean diet predicts 9-year changes in episodic memory in an adult lifespan sample of Americans

**DOI:** 10.1177/13872877251320861

**Published:** 2025-03-17

**Authors:** Kelly RB Parker, Ryan McGrath, Yeong Rhee, Jeremy Hamm

**Affiliations:** 1 Motivation and Healthy Adult Development Lab, North Dakota State University, Fargo, ND, USA; 2 Healthy Aging North Dakota, North Dakota State University, Fargo, ND, USA; 3 Department of Health, Nutrition, and Exercise Sciences, North Dakota State University, Fargo, ND, USA

**Keywords:** Alzheimer's disease, cognitive aging, cognitive decline, healthy lifestyle, longitudinal study, nutrition assessment

## Abstract

**Background:**

The Mediterranean Diet (MD) is well-studied for slowing cognitive declines. Few studies have examined how a Western MD (wMD) may impact cognitive function.

**Objective:**

This study examined whether a wMD predicted less cognitive decline over 9 years in a national sample of American adults. The measures were episodic memory (EM) and executive functioning (EF) at baseline and 9 years follow-up.

**Methods:**

This is a secondary analysis of the Midlife in the United States Study (MIDUS), using a longitudinal cohort design with cross-sectional dietary data. Participants in this study had data from Waves 2 and 3 of MIDUS (*n *= 833, 46 ± 12 years; 45% male). Regression analyses tested whether wMD adherence predicted 9-year changes in EM and EF. Moderator analyses determined whether the relationship between wMD, EM, and EF differed across sociodemographic characteristics.

**Results:**

wMD score at Wave 2 predicted attenuated declines in EM 9 years later (β = 0.06, *p *= 0.04). The association between wMD and EM was not moderated by age, sex, race, education, or income and thus is consistent across sociodemographic subpopulations. wMD did not predict EF (fully adjusted wMD β = 0.00, *p *= 0.86). Contextualized effect sizes showed that individuals who strongly adhered to the wMD (+1 SD) experienced ∼50–60% less decline in 9-year EM when compared to those with average adherence.

**Conclusions:**

A wMD was related to slowed EM declines across sociodemographic populations in a national U.S. sample. Education is needed about healthful dietary habits, including increased fruit and vegetable intake.

## Introduction

The Mediterranean Diet (MD) is a widely studied behavioral factor that has been proposed to buffer the cognitive declines associated with Alzheimer's disease and Alzheimer's disease-related dementias (AD/ADRD). As longevity increases, research on the role of modifiable health behaviors such as MD to reduce risk and delay onset of AD/ADRD is of paramount importance given that 1 in 3 adults over the age of 85 develop some form of dementia.^1^ Additionally, about 4% of adults age 65 and older have received a dementia diagnosis.^2^ The MD focuses on increased consumption of fruits, vegetables, whole grains, calcium-rich dairy foods, olive oil, and seafood, while reducing consumption of added sugars, heavily processed foods, and saturated fat from red meat.^3^ This dietary pattern has been linked to better health and cognitive outcomes during aging.^4–7^

A previous study by Tangney et al. (2011) showed that adherence to the MD in old age reduced the risk of cognitive impairment over a 7-year follow-up.^8^ Further research by Tangney et al. (2014) extended this earlier work by showing that MD was linked to slower declines in more sensitive indicators of cognitive aging involving preserved episodic memory.^9^

Another comparable investigation found that this relationship held over extended periods of time (12–17 years) in an older Mediterranean population.^10^ Scarmeas et al. (2009) further highlighted the benefits of the MD in older Americans with his novel scoring tool by demonstrating a reduction in the progression towards mild cognitive impairment and dementia amongst individuals adhering to an MD eating pattern more closely than their peers.^7^ Although this previous work provides strong evidence for the protective role of MD on cognitive function, most studies focus solely on older adults and coarse measures of cognitive function that are less sensitive to the small changes that individuals may experience over shorter time intervals as part of the normal aging process.^11–16^ Critical open questions thus remain about the role of MD in young and middle-aged adults who may gain the greatest long-term benefit from adopting dietary changes earlier in the lifespan for reducing the risk of early onset dementia.

Other studies have very brief periods of follow-up sometimes measured in months rather than years, likely due to the difficulties of maintaining a longitudinal cohort.^17^ Few studies have examined whether the relationship between MD and cognitive function holds in a large, national sample of adults from the United States (U.S.) over an extended period of time. Moreover, almost no studies have examined this relationship in an adult lifespan sample, with the exception of the Personality & Total Health Through Life cohort (PATH).^15,16^ Although the PATH cohort provided valuable information, the key focus of the research was on a modification of the MD, the MIND diet, which combines the Dietary Approaches for Stopping Hypertension, or DASH diet, with the MD. This modification includes more use of low-fat dairy foods and adds a focus on reduction of sodium intake along with increasing calcium and potassium intake.

While the MIND diet has health benefits, its stringent guidelines and required tracking and label reading make it challenging to follow over extended time periods, and therefore less feasible as it may require a substantial dietary overhaul. For example, previous work has found that Americans largely under-consume fruits, vegetables, whole grains, calcium-rich dairy foods and seafood, and overconsume red and processed meats, refined grains, added sugars and especially sugar-sweetened beverages (SSBs).^18^ Furthermore, American adults may not consume olive oil as often as other plant-based oils.^19^ This combination of factors suggests that a modification of the MD that still addresses key tenets of dietary quality such as fruit and vegetable intake while being more lenient in regards to protein sources may be helpful in moving American adults toward a more healthful diet.

Using national MIDUS data, our study examined the role of such a modified, Western MD (wMD), in buffering against longitudinal declines in two key domains of cognitive functioning that are sensitive to early age-related losses: episodic memory and executive functioning.^20^ The wMD is based on the scoring tool utilized by the PREDIMED study, with modifications to account for current dietary habits and recommendations in the U.S. as identified in the Dietary Guidelines for Americans.^18,21^ The wMD focused on the potential benefits of increased consumption of fruits, vegetables and fish, and decreased consumption of sugar-sweetened beverages and fast food.

The wMD allows for a lenient consumption of dairy, lean meat and poultry, as well as up to 3 servings per week of red, fatty, or processed meats, and for any non-animal protein food to be included as a source of lean proteins. Increased allowance of dairy products is based on previous findings that the protein-calcium matrix found in milk, cheese, and especially yogurt may be beneficial to cognitive health in the aging process along with well-documented reductions in sarcopenia and osteoporosis.^22,23^ However, low-fat and fat-free versions are generally recognized as being more healthful due to the reduction in saturated fat content.^24^ Even so, with the wide availability of low-fat and fat-free dairy products in the U.S., this may be a less expensive and less processed source of protein than other protein-rich foods. Additionally, while the amount of red, fatty, and/or processed meats (including fried chicken, ribs, and sausage) allowed under the wMD are higher than recommended in the traditional MD, this allowance is still much lower than the nearly 8 servings of red, fatty, or processed meats typically consumed by an American adult, and is less than the 1 serving per day allowed by the PREDIMED guide.^21,25^ Lean protein sources included white meat chicken or poultry, lean beef or lean pork based on data available in MIDUS. Furthermore, wine is not considered in this calculation due to disagreement as to whether it should be recommended, and olive oil was excluded due to lack of available data. We did, however, include a point for consuming fast food once a week or less often.

These modifications in the wMD retain the benefits of a diet rich in fruits, vegetables, whole grains, and lean protein sources and closely aligns with other healthful dietary patterns associated with reduced risk of several morbidities.^4,7,11,13,21,23,26–30^ We examined whether this more feasible wMD was associated with longitudinal episodic memory and executive functioning over a 9-year period in a national cohort of Americans across the adult lifespan. We also examined the extent to which associations between the wMD and cognitive functioning differed across (were moderated by) key sociodemographic characteristics.

## Methods

The National Midlife in the United States (MIDUS) study was reviewed and approved by the University of Wisconsin-Madison, and its methods have been described elsewhere.^31^ Briefly, the MIDUS study began in 1995 with 7108 participants aged 25–74 at baseline who were followed every 9 years until mortality or study dropout. The MIDUS study focuses on the health and wellbeing of adults throughout the adult lifespan with a biopsychosocial model, and has integrated biomarkers, genetics, and occupational data to its original survey sampling design for a subset of the participants. The Brief Test of Adult Cognition by Telephone (BTACT) was added in Wave 2 as Project 3.^32^ A food frequency questionnaire was also added at Wave 2 as part of MIDUS Project 4 (the Biomarker project).^33^ Participants were included if they participated in the Biomarker Project at Wave 2, provided complete dietary data at Wave 2, and provided cognitive data at Waves 2 and 3.

### Participants

Those providing only dietary data (*n *= 1053: 55 ± 12 years of age; 45% male) were largely similar to those who also provided at least some cognitive functioning data (*n*_EM _= 860: 54 ± 11 years of age; 44% male). All individuals lived in the United States and were initially contacted using a random digit dialing system to recruit a national sample. More information about MIDUS and its participants is available elsewhere.^34–36^ MIDUS data is publicly available for download after registration.^37^

### Western Mediterranean (wMD) diet score calculation

The MIDUS Medical History Questionnaire was reviewed for typical dietary practices of respondents. This questionnaire was administered by trained interviewers when the participants came to the site for their clinical assessments, including a blood draw, urine, and saliva samples.^33^ These data included servings of each of the variables and their frequency (daily, weekly, monthly, etc.). Therefore, each food item was recalculated to compare directly against the frequency of consumption that would align with the commonly recognized MD (e.g., frequency of dairy consumption on a daily basis, fish and other protein foods’ consumption on a weekly basis, etc.) and the 2020–2025 Dietary Guidelines for Americans (USDGA). The frequency of consumption was then compared against the UK's cardiac rehabilitation Mediterranean Diet Score Tool, a variation on the PREDIMED Mediterranean Diet Scoring Tool, with a binary score of 1 or 0 for each item, so that a score of 1 indicated compliance.^38–40^ Appropriate portion sizes and frequencies were determined based on the UK's Cardiac Rehabilitation Mediterranean Diet Score Tool in conjunction with the average serving amounts for the USDGA (e.g., 1 cup of fruits or vegetables equated to 1 serving and 1 ounce of whole grains equated to 1 serving). Scores were summed and a higher overall score indicated greater compliance.^38^

Because the MIDUS questionnaire did not include a separate question for fat sources, olive oil was not included in the wMD score calculation. The fat content of milk was not measured as part of the MIDUS food frequency questionnaire. Wine consumption was excluded from the wMD score because there is disagreement about the potential health benefits of wine, and the 2020–2025 Dietary Guidelines for Americans does not recommend wine consumption for people who do not already consume alcohol.^18^ The score was further modified to include a point for fast food frequency of less than or equal to once per week, which is less than the average for Americans.^41,42^ Due to these changes, the possible scores for the wMD ranged from 0 to 11. Items incorporated included servings of milk, yogurt, cheese, sugar-sweetened beverages, servings of fruits and vegetables, servings of whole grains, fish, beef or other fatty and processed meats, lean meat, and non-meat proteins, as well as fast food frequency. Adjustments were made to the allowable number of servings of lean meat, red, fatty, or processed meats, and total amount of dairy products in order to address current dietary habits of Americans and make adherence to the wMD more attainable. Table 1 includes the scoring criteria for the 11 items. The wMD exhibited a moderate-to-strong correlation with the previously validated MIDUS-Healthy Eating Index (Pearson's *r *= 0.69, *p *< 0.001).^43^

**Table 1.** Scoring criteria for Western Mediterranean diet.

Food item	Frequency	Amount**^a^**	Score if criteria is met/not met
Milk	Daily	>0 - ≤ 3 servings	1/0
Yogurt	Daily	>0 - ≤ 3 servings	1/0
Cheese	Daily	>0 - ≤ 2 servings	1/0
Sugar-sweetened Beverages	Daily	<1 serving	1/0
Whole Grains	Daily	≥3 servings	1/0
Fruits and Vegetables	Daily	≥5 servings	1/0
Fish	Weekly	≥3 servings	1/0
Red, Fatty, or Processed Meats	Weekly	≤ 4 servings	1/0
Lean Meats	Weekly	>3 serving	1/0
Non-Meat Proteins	Weekly	≥3 servings	1/0
Fast Food	Weekly	≤1 servings	1/0

^a^ Items that are indicated as having >0 could have any amount greater than 0 up to the indicated limit due to the nature of calculations, e.g., 0.01 servings of milk per day would qualify for a full point. For convention's sake, all items were rounded to 2 decimal places during calculation.

### Cognitive functioning

The Brief Test of Adult Cognition by Telephone (BTACT) was used to assess episodic memory and executive functioning at Waves 2 and 3.^44,45^ Previous research has found the BTACT to be reliable, valid, and sensitive in measuring episodic memory and executive functioning in middle-aged and older adults.^20,32,44,46–48^ A detailed summary of the BTACT can be found elsewhere.^20,32^

In brief, the BTACT battery included two cognitive tests for episodic memory and five for executive functioning.^32^ Episodic memory was assessed using immediate and delayed recall tasks with a free recall of 15 words. Executive functioning was assessed using measures of inductive reasoning (completing patterns in a number series), category verbal fluency (number of animal names produced in one minute), processing speed (number of digits produced counting backwards from 100 in 30 s), working memory (backward digit span), and attention switching and inhibitory control (Stop and Go Switch Task). For the executive functioning measure, a recommended filter was used to retain data for only those participants with valid scores on the Stop and Go Switch Task.^32,44^ Valid scores were those with no technical malfunctions, where the participant understood the task and was not distracted by external events. It should be noted that the sample size differs between the episodic memory and executive functioning datasets due to instances of nonvalid data in the executive functioning tasks for the Stop and Go Switch Task. As described in previous studies,^46,49^ measures of episodic memory and executive functioning were calculated by averaging the standardized values of their respective subtests at each wave.^20^

### Sociodemographic covariates and moderators

Age, sex, race, and socioeconomic status (SES) are well-established correlates of cognitive functioning and were included as covariates in the analyses.^20,50–52^ Age in years was assessed at Wave 2. Sex (1 = male, 2 = female) and race (0 = White, 1 = non-White) were assessed at Wave 1. Three self-reported measures of SES were assessed at Wave 2, including level of formal education completed, total household income in U.S. dollars, and perceived SES using the reverse-coded MacArthur Scale of Subjective Social Status. A composite SES score was created from these variables and utilized as in previous studies.^46,53^ Instrumental activities of daily living limitations (IADLs) as measured at wave 2 (baseline) were likewise included. Participants reported the extent to which health limited their ability to perform 7 IADLs using a 4-point scale (1 = a lot, 4 = not at all; e.g., carrying groceries, climbing several flights of stairs, etc.). Scores were reverse coded so that higher scores reflected greater functional limitations.

### Statistical analyses

Autoregressive, ordinary least squares (OLS) regression models were employed to test our hypothesis that a wMD would prospectively predict longitudinal changes in episodic memory and executive functioning. Step 1 models assessed the extent to which wMD at baseline (Wave 2) predicted 9-year changes in each indicator of cognitive functioning while controlling for age, sex, race, socioeconomic status (SES), and baseline (Wave 2) levels of the outcome measures (i.e., autoregressive effects). This autoregressive approach is recommended over a raw change score approach, which may bias estimates from pre-existing baseline differences in the outcome measures.^54^ Step 2 models assessed the extent to which key sociodemographic characteristics including age, sex, race, and SES moderated the associations between wMD and longitudinal changes in cognitive functioning. Step 3 models assessed the extent to which wave 2/baseline IADLs influenced the relationship between wMD and cognitive functioning. All models were run using SPSS version 27.0 using an alpha level of significance at 5% or 0.05.^55^ Supplemental analyses were conducted to examine more traditional versions of the MD along with individual items’ average daily consumption (see Supplemental Material).

## Results

As is typical of longitudinal studies, participants who completed wave 3 were more likely to be younger than those who dropped out between waves 2 and 3, though the effect size was relatively small (*d *= 0.37, p < 0.001).^56^ Descriptive analyses indicated the average wMD score at Wave 2 of the MIDUS biomarker project was 5.95 ± 1.73 on an 11-point scale with scores ranging from 1–11 and a median score of 6. The most commonly received point was for lean meat consumption, and the least commonly received point was for fish consumption with only 86 participants (8.2%) receiving the point. Table 2 contains a summary of the percentages of the sample who received a point for each area of intake from all participants who provided dietary intake data in the food frequency questionnaire (N = 1053).

**Table 2.** Proportions of participants meeting Western Mediterranean diet guidelines (N = 1053).

Food item	Percentage	Frequency
Milk	70.1%	738
Yogurt	46.0%	484
Cheese	67.1%	707
Sugar-sweetened Beverages	67.3%	709
Whole Grains	23.9%	252
Fruits and Vegetables	20.9%	220
Fish	8.2%	86
Red, Fatty, or Processed Meats	90.9%	957
Lean Meat	95.0%	1000
Non-Meat Protein	56.5%	595
Fast Food	51.4%	541

Our regression analyses revealed that the wMD predicted longitudinal changes in episodic memory in both the raw, unadjusted model (β = 0.08, SE = 0.030 *p *= 0.011), and in the fully adjusted model (β = 0.06, SE = 0.030, *p *= 0.037), such that greater adherence to the wMD prospectively predicted less decline in episodic memory over the 9-year follow-up period. Table 3 summarizes the results for episodic memory in the unadjusted and adjusted models, while Table 4 summarizes the findings for executive functioning. Adherence to the wMD did not predict longitudinal changes in executive functioning in either the unadjusted or adjusted models (*p *= 0.246).

**Table 3.** Main effect model regression coefficients for 9-year regressed changes in episodic memory (n = 833).

	Model 1a		Model 1b		Model 1c	
	β	*b (SE)*	β	*b (SE)*	β	*b (SE)*
Baseline EM	0.52**	0.60 (0.03)	0.39**	0.45 (0.04)	0.39**	0.45 (0.04)
Baseline wMD	0.08*	0.04 (0.02)	0.07*	0.04 (0.02)	0.06*	0.04 (0.02)
Age			−0.22**	−0.02 (<0.01)	−0.21**	−0.02 (<0.01)
Sex (Female)			0.18**	0.35 (0.06)	0.19**	0.37 (0.06)
Race (Minority)			−0.01	−0.05 (0.12)	−0.02	−0.06 (0.12)
SES			0.10**	0.14 (0.04)	0.08**	0.12 (0.04)
Baseline (w2) IADL Limitations					−0.06*	−0.08 (0.04)

Models were built using a step-wise approach with an unadjusted model that predicted the outcome (EM) from only the Mediterranean diet. Each subsequent model was adjusted for potential covariates and confounders, including demographics and other health limitations. EM: episodic memory; wMD: Western Mediterranean diet; SES: socioeconomic status; IADL: Instrumental activities of daily living. β coefficients are standardized. *b* coefficients are unstandardized. *indicates the effect size is significant at p < 0.05; **indicates significance at p < 0.01.

**Table 4.** Main effect model regression coefficients for 9-year regressed changes in executive functioning (N = 796).

	Model 1a		Model 1 b		Model 1c	
	Β	*b (SE)*	β	*b (SE)*	β	*b (SE)*
Baseline EF	0.78**	1.16 (0.03)	0.68**	1.01 (0.04)	0.65**	0.75 (0.03)
Baseline wMD	−0.03	−0.02 (0.01)	0.01	0.01 (0.01)	0.00	0.00 (0.01)
Age			−0.22**	−0.02 (0.00)	−0.21**	−0.01 (0.00)
Sex (Female)			−0.05*	−0.09 (0.04)	−0.05*	−0.07 (0.03)
Race (Minority)			−0.01	−0.04 (0.08)	−0.02	−0.06 (0.06)
SES			0.06*	0.08 (0.03)	0.04*	0.04 (0.02)
Baseline (w2) IADL Limitations					−0.06*	−0.05 (0.02)

Models were built using a step-wise approach with an unadjusted model that predicted the outcome (EF) from only the Mediterranean diet. Each subsequent model was adjusted for potential covariates and confounders, including demographics and other health limitations. EF: executive function; wMD: Western Mediterranean diet; SES: socioeconomic status; IADL: Instrumental activities of daily living. β coefficients are standardized. *b* coefficients are unstandardized. *indicates the effect size is significant at p < 0.05; **indicates significance at p < 0.01.

**Figure 1.**
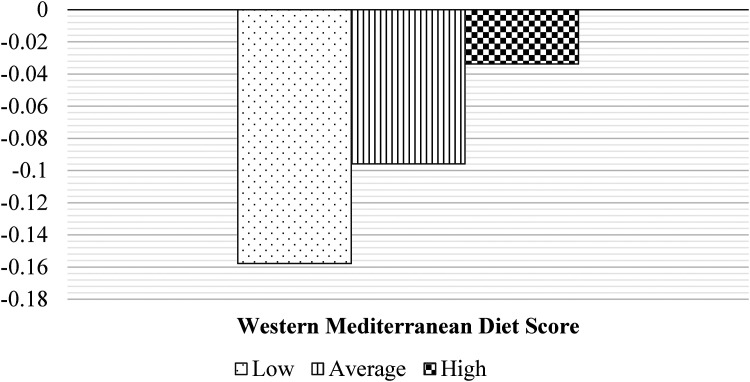
Western Mediterranean diet predicts 9-year declines in episodic memory (N = 833)^†^.

^†^Low Western Mediterranean Diet Score is 1 standard deviation below the mean score; Average is the mean score, and high is 1 standard deviation above the mean score.

To contextualize the main effects on episodic memory, we generated predicted values (PVs) that adjusted for raw, average sample declines of −0.10 units over the 9-year follow-up. Small but meaningful differences emerged for participants who scored a standard deviation below the mean on wMD (PV = −0.16), who had average wMD (PV = −0.10), and who scored a standard deviation above the mean on wMD (PV = −0.03). These estimates are summarized in Figure 1 and suggest that rates of decline in episodic memory were reduced by approximately 70% (−0.10 versus −0.03) for individuals who strongly adhered to the wMD relative to those with average adherence to the wMD.

Moderation models tested whether the association between wMD and longitudinal cognitive functioning depended on key sociodemographic characteristics including age, sex, race, education, and income. Results from these analyses yielded no significant interactions for episodic memory (age *p *= 0.341, sex *p *= 0.341, race *p *= 0.308, education *p *= 0.783, income *p *= 0.176). The same pattern emerged for executive functioning, such that no significant interactions were observed (age *p *= 0.982, sex *p *= 0.765, race *p *= 0.339, and education *p *= 0.547, income *p *= 0.528) indicating that greater adherence to the wMD had benefits for longitudinal episodic memory that were consistent across subpopulations that varied in age, sex, race, income, and education.

## Discussion

The principal findings from our investigation revealed that a wMD may provide protective effects for cognitive function in an adult lifespan sample in the U.S. Previous studies have found that high adherence to an MD diet is associated with lower AD pathology and risk,^7,13,14^ and slower declines in episodic memory.^11,12^ However, few studies examined the role of a wMD in an adult lifespan sample in the U.S. with a long-term follow-up. Even less dietary research has been conducted using sensitive measures of cognitive change such as the BTACT rather than more broad-strokes tools such as the Telephone Interview for Cognitive Status, Mini-Mental State Exam or the Montreal Cognitive Assessment which are less sensitive to subclinical declines in cognitive functioning that may occur earlier in the life course.^56^ A few of the studies that have examined these more sensitive measures include the Chicago Health and Aging Project,^9^ the Maine-Syracuse Longitudinal Study,^57^ as well as the Lothian Birth Cohort 1936^30^ and others.^7,16,58–60^ However, all of these studies were based solely on older adults at their outset while our study examined individuals across the adult lifespan.

Our findings showed that executive functioning was not impacted by the wMD score. This could be due to a number of factors, such as no specific inclusion of olive oil of any type in our measure due to lack of available data on consumption in MIDUS. Monounsaturated fats found in olive oil, and extra virgin olive oil in particular, have previously been linked to improved executive functioning in older adults who follow the MD eating pattern.^59,61^ Other factors beyond diet may have influenced the null finding, including a strong autoregressive effect on executive functioning, making it less able to be impacted by behavioral changes. Episodic memory may therefore be more noticeably attenuated by dietary differences.

Numerous mechanisms have been proposed for as an explanation for the benefits of the MD eating pattern, including reductions in oxidative stress and inflammatory biomarkers from the high intake of antioxidants and polyphenols from fruits, vegetables, and whole grains.^24,61^ Other social and behavioral factors of the Mediterranean lifestyle, including meals with family and friends and regular incorporation of physical activity in daily life have also been proposed. These health behaviors are likely to lead to improvements in cardiovascular function and better control of chronic conditions such as diabetes, thereby reducing the risk of Alzheimer's disease and related dementias. The source of the benefits is likely a combination of all of these factors, and future studies should attempt to parse out how much each of these modifiable factors is contributing to cognitive benefits.

While our study is based on a prospective cohort design, a reasonably large national sample, and a 9-year lag between cognitive data collections, it is not without limitations. Firstly, as is typical in large-scale, longitudinal field studies, MIDUS measures of diet were based on self-reported intakes using a food-frequency questionnaire.^62^ While food frequency questionnaires measure habitual intake, it is possible that individuals’ diets changed over the course of the study. Furthermore, people tend to underestimate their portion sizes and energy intake, so it is possible that the portion sizes indicated by the food frequency questionnaire are lower than what was actually consumed.^63^ Future research is needed using behaviorally assessed diet. While we controlled for age, sex, race, and socioeconomic status, it is possible that another, unmeasured variable impacted the results, including differences between those who did not participate in either the biomarker project or wave 3 of MIDUS. It is possible that survival bias or other factors may have influenced our results, as participants were lost to follow-up between waves. Further research is also needed in racially and socioeconomically diverse populations whose culinary methods and dietary habits may impact their adherence to the wMD. Beyond differences of culture, differences in genetic makeup may contribute to different nutrient utilization, which could result in some populations experiencing different or even no benefits from the wMD.

Our study is among the first to examine a Western adaptation of the MD that may be beneficial for adults in the U.S. Specifically, this study examined the role of the 2020–2025 Dietary Guidelines for Americans’ and the UK's Cardiac Rehabilitation Mediterranean Diet Score Tool's recommended levels of whole grains (≥3 servings per day), vegetables (>3 servings per day), fruits (>2 servings per day), and lean protein foods, with the addition of dairy intake, including milk, yogurt, and cheese (>0 to ≤3 servings per day = 1 point each). Practically, this wMD may reflect a valuable clinical tool for community health workers and physicians who provide frontline recommendations and advice to the population at large and particularly to those at risk of adverse health events related to poor diet and exercise. The finding that the wMD provides protective benefits for episodic memory across sociodemographic subpopulations highlights the value of dietary recommendations considering health benefits not only for avoiding chronic diseases of metabolism, but also for slowing cognitive decline.

The present findings identify the benefits of following a Western version of the MD, which can be achieved without assessing intake of olive oil. Making small-scale changes in dietary habits can result in small, but clinically meaningful improvements in health and slowed cognitive decline over time.^64^ Focusing on minor changes such as increasing fruit and vegetable consumption or reducing sugar-sweetened beverage intake even by small amounts may help patients to maintain health behaviors over longer periods of time than more drastic changes, thus resulting in better long-term health. As evidenced by our study, a single standard deviation's increase in wMD adherence resulted in a 2/3 slower decline in episodic memory than an average consumer of the wMD.

This study is strengthened by several factors that include an adult lifespan sample, extended follow-up period, and autoregressive analytic approach that controlled for preexisting differences in cognitive functioning. However, it is limited by its observational nature and the use of a semi-quantitative food frequency questionnaire for dietary intake assessment. Even so, longitudinal cohort studies are observational by design, and food frequency questionnaires have been shown to produce a reasonable estimate of typical dietary intake within these epidemiological studies.^65,66^ Another possible confounder is the collection of dietary intake data in any form, as participants may report intakes reflecting what they view as being healthy or desirable. The tool used for this particular wMD has not previously been employed, though it was loosely based on an adaptation of the PREDIMED study used by the UK's Cardiac Rehabilitation programs and the United States Dietary Guidelines for Americans recommendations.^18,21,38^ Further, it was moderately correlated with the MIDUS Healthy Eating Index, suggesting a reasonable degree of confidence in our findings.^67^ It should, however, be noted that the wMD differs significantly from previous versions of the MD or MIND diet, and utilized a different measurement strategy based on available data.

This study extends the literature by proposing a simple and feasible alternative to the MD that is based on the United States Dietary Guidelines for Americans. It provides initial evidence that the relationship between MD and cognitive function over an extended period is not moderated by sociodemographic differences in this sample. This suggests that the wMD may be beneficial for all adults, regardless of sociodemographic characteristics.

### Conclusions and implications

The MD continues to show promise as a possible means for slowing cognitive decline in the aging process. These findings provide evidence that the wMD benefits cognitive aging across subpopulations of American adults as all demographic groups showed similar benefit. This novel finding suggests that small-scale dietary differences in individuals are associated with improved cognitive trajectories regardless of race, sex, or age with a 1 standard deviation increase in wMD being associated with a 2/3 slower episodic memory decline than average consumers. Future studies should examine how changes in wMD adherence may precede changes in cognitive function to help determine directionality and better clarify the strength of this relationship in various populations within the United States and globally. Clinicians should encourage their patients to adhere to a MD or other healthful eating pattern when possible, with special emphasis on consuming more fruits, vegetables, nonmeat protein sources, and fish, and fewer sugar-sweetened beverages and less fast food as these habits have consistently shown beneficial relationships with overall and cognitive health.

## Supplemental Material

sj-docx-1-alz-10.1177_13872877251320861 - Supplemental material for Western Mediterranean diet predicts 9-year changes in episodic memory in an adult lifespan sample of Americans Supplemental material, sj-docx-1-alz-10.1177_13872877251320861 for Western Mediterranean diet predicts 9-year changes in episodic memory in an adult lifespan sample of Americans by Kelly RB Parker, Ryan McGrath, Yeong Rhee and Jeremy Hamm in Journal of Alzheimer's Disease
