# Supplementary material for: Western Mediterranean diet predicts 9-year changes in episodic memory in an adult lifespan sample of Americans
Source: J Alzheimers Dis. 2025 Mar 17;104(3):943–52. doi: 10.1177/13872877251320861 (PMC12087284; doi:10.1177/13872877251320861)
Supplement: sj-docx-1-alz-10.1177_13872877251320861 - Supplemental material for Western Mediterranean diet predicts 9-year changes in episodic memory in an adult lifespan sample of Americans [file sj-docx-1-alz-10.1177_13872877251320861.docx]

**Supplemental Material**

**Western Mediterranean diet predicts 9-year changes in episodic memory in an adult lifespan sample of Americans**

**Supplemental Analyses**

To identify whether the higher allowance of dairy was potentially masking an effect for executive functioning, a revised wMD scale utilizing a single item for dairy was used in place of the original wMD scale. This resulted in a 9-point wMD (w9MD) measure with a single dairy item in place of separating the 3 categories of dairy in the original measure. We also examined whether stronger effects for episodic memory were observed for the w9MD. Supplemental analyses utilizing consumption of each individual item in place of the composite score were also conducted to identify the key dietary sources of the observed effects. Finally, based on results from the supplemental analyses a mini-wMD scale was created using only 2 items from the original-wMD scale.

**Supplemental Results**

**Supplemental Table 1.** Main Effect Model Regression Coefficients for 9-year Regressed Changes in Executive Functioning using 9-point wMD (w9MD) (N = 796)

|  | Model S1a | | Model S1 b | | Model S1c | |
| --- | --- | --- | --- | --- | --- | --- |
|  | β | *b (SE)* | β | *b (SE)* | β | *b (SE)* |
| Baseline EF | 0.76** | 0.88 (0.03) | 0.66** | 0.77 (0.03) | 0.66** | 0.76 (0.03) |
| Baseline w9MD | -0.06* | -0.03 (0.01) | -0.02 | -0.01 (0.01) | -0.02 | -0.01 (0.01) |
| Age |  |  | -0.21** | -0.01 (0.00) | -0.20** | -0.01 (0.00) |
| Sex (Female) |  |  | -0.05* | -0.07 (0.03) | -0.04 | -0.06 (0.03) |
| Race (Minority) |  |  | -0.01 | -0.04 (0.07) | -0.02 | -0.05 (0.07) |
| SES |  |  | 0.05* | 0.05 (0.02) | 0.04 | 0.04 (0.02) |
| Baseline (w2) IADL Limitations |  |  |  |  | -0.06** | -0.06 (0.02) |
| Models were built using a step-wise approach with an unadjusted model that predicted the outcome (EF) from only the Mediterranean diet. Each subsequent model was adjusted for potential covariates and confounders, including demographics and other health limitations. EF: executive function; w9MD: Western Mediterranean diet 9-point version; SES: socioeconomic status; IADL: Instrumental activities of daily living. β coefficients are standardized. *b* coefficients are unstandardized. *indicates the effect size is significant at p<0.05; **indicates significance at p<0.01. | | | | | | |

**Supplemental Table 2.** Main Effect Model Regression Coefficients for 9-year Regressed Changes in Episodic Memory using 9-point wMD (w9MD) (N = 796)

|  | Model S1a | | Model S1 b | | Model S1c | |
| --- | --- | --- | --- | --- | --- | --- |
|  | β | *b (SE)* | β | *b (SE)* | β | *b (SE)* |
| Baseline EM | 0.52** | 0.60 (0.03) | 0.40** | 0.46 (0.04) | 0.40** | 0.46 (0.04) |
| Baseline w9MD | 0.05 | 0.03 (0.02) | 0.05 | 0.03 (0.02) | 0.04 | 0.03 (0.02) |
| Age |  |  | -0.22** | -0.02 (0.00) | -0.21** | -0.02 (0.00) |
| Sex (Female) |  |  | 0.18** | 0.35 (0.06) | 0.19** | 0.37 (0.06) |
| Race (Minority) |  |  | -0.01 | -0.03 (0.12) | -0.01 | -0.04 (0.12) |
| SES |  |  | 0.10** | 0.14 (0.04) | 0.08** | 0.12 (0.04) |
| Baseline (w2) IADL Limitations |  |  |  |  | -0.06* | -0.08 (0.04) |
| Models were built using a step-wise approach with an unadjusted model that predicted the outcome (EM) from only the Mediterranean diet. Each subsequent model was adjusted for potential covariates and confounders, including demographics and other health limitations. EM: episodic memory; w9MD: Western Mediterranean diet 9-point version; SES: socioeconomic status; IADL: Instrumental activities of daily living. β coefficients are standardized. *b* coefficients are unstandardized. *indicates the effect size is significant at p<0.05; **indicates significance at p<0.01. | | | | | | |

**Supplemental Table 3.** Main Effect Model Regression Coefficients for 9-year Regressed changes in Episodic Memory using Single Item Estimated Servings

|  | β | *b (SE)* | |
| --- | --- | --- | --- |
| Daily Milk | -0.01 | -0.01 (0.03) | |
| Daily Cheese | 0.04 | 0.03 (0.02) | |
| Daily Yogurt | -0.02 | -0.03 (0.06) | |
| Daily Dairy (total) | 0.02 | 0.01 (0.02) | |
| Daily Fruits and Vegetables | 0.08** | 0.09 (0.04) | |
| Daily Whole Grains | 0.00 | 0.00 (0.04) | |
| Daily Sugar-sweetened Beverages | -0.00 | -0.00 (0.03) | |
| Weekly Fish | 0.01 | 0.02 (0.03) | |
| Weekly High-Fat Meats | 0.02 | 0.02 (0.03) | |
| Weekly Lean Meats | 0.03 | 0.03 (0.03) | |
| Weekly Non-meat Proteins | 0.07** | 0.07 (0.03) | |
| Weekly Fast Food | 0.01 | 0.01 (0.03) | |
| All items were added to a model controlling for baseline (w2) episodic memory (EM), age, sex, race, socioeconomic status, and limitations of instrumental activities of daily living (IADLs) at baseline (w2). Race was nonsignificant in all models; EM, age, sex, SES, and IADLs were significant in all models. *indicates the effect is significant at p<0.05, **indicates significance at p<0.01. | | |  |

**Supplemental Table 4.** Main Effect Model Regression Coefficients for 9-year Regressed changes in Episodic Memory using mini-MD (mMD) Scale.

|  | β | *b (SE)* |
| --- | --- | --- |
| Baseline EM | 0.39** | 0.46 (0.04) |
| mMD | 0.07* | 0.10 (0.04) |
| Age | -0.20** | -0.02 (0.00) |
| Sex (Female) | 0.19** | 0.38 (0.06) |
| Race (Minority) | -0.01 | -0.04 (0.12) |
| SES | 0.08** | 0.12 (0.04) |
| Baseline (w2) IADL Limitations | -0.06* | -0.08 (0.04) |
| The miniMD (mMD) consisted of 2 items, average daily servings of fruits and vegetables and weekly servings of non-meat protein foods. *indicates the effect is significant at p<0.05; **indicates significance at p<0.01. | | |
